# From Folk Taxonomy to Species Confirmation of *Acorus* (Acoraceae): Evidences Based on Phylogenetic and Metabolomic Analyses

**DOI:** 10.3389/fpls.2020.00965

**Published:** 2020-06-24

**Authors:** Zhuo Cheng, Hang Shu, Shuang Zhang, Binsheng Luo, Ronghui Gu, Ruifei Zhang, Yuanyuan Ji, Feifei Li, Chunlin Long

**Affiliations:** ^1^College of Life and Environmental Sciences, Minzu University of China, Beijing, China; ^2^Key Laboratory of Ethnomedicine (Minzu University of China), Ministry of Education, Beijing, China; ^3^State Key Laboratory of Environmental Criteria and Risk Assessment, Chinese Research Academy of Environmental Sciences, Beijing, China

**Keywords:** *Acorus*, chemotaxonomy, DNA barcoding, ethnobotany, parataxonomy, metabolomics, species identification

## Abstract

Plants in *Acorus* have been used as herbal medicine by various linguistic groups for thousands of years. Arguments of taxonomy of *Acorus* among scientists resulted in confusions and misuses of *Acorus* plants. The present study used different methods to investigate the classification of the genus, based on folk taxonomy. The relationships among *Acorus* species were revealed through phylogenetic analyses by constructing the Maximum Likelihood and Bayesian phylogenetic analyses based on sequences of two chloroplast regions (*trnL-trnF* and *rbcL*). All samples named with two so-called synonyms, *Acorus macrospadiceus* (Yamam.) F. N. Wei and Y. K. Li and *Acorus tatarinowii* Schott collected from different habitats, were clustered into separate groups, which revealed that they represented two independent species. Multivariate statistical analysis of metabolites from different *Acorus* populations were carried out based on UPLC-QTOF-MS data. Three independent analysis, principal component analysis, heat-map analysis, and hierarchical cluster analysis, showed that *A. macrospadiceus* and *A. tatarinowii* are different from two recognized species in the genus, *A. calamus* L. and *A. gramineus* Aiton. The results of phylogenetics and chemotaxonomy, together with morphological and ecological evidences, were consistent with traditional knowledge of local people related to *Acorus* taxa, which proved the significance of parataxonomy. Multiple evidences including morphological, ecological, folk taxonomic, phylogenetic, and chemical taxonomic results suggested that there are four species in the genus *Acorus*.

## Introduction

The genus *Acorus*, belonging to the family Acoraceae, is mainly distributed from northern temperate to subtropical regions (Li H. et al., 2010; Feng et al., 2015). It was once a member of Araceae and regarded as a relatively primitive group in the family before redesignation as Acoraceae (Li H. et al., 2010). Although species in this genus have multiple values including medicinal, nutritional, ornamental, cultural, economic, and ecological uses (Motley, 1994; Shu et al., 2018a), the taxonomy of *Acorus* remains unclear (Pan et al., 2002). Four species namely *A. calamus* L., *A. gramineus* Aiton, *A. tatarinowii* Schott, and *A. rumphianus* S. Y. Hu were recorded in *Flora Reipublicae Popularis Sinicae* (FRPS) (Li, 1979). However, only two species, *A. calamus* (and varieties) and *A. gramineus*, were accepted in *Flora of China* (FOC) (Li et al., 2010), *the Plant List* (http://www.theplantlist.org/) and *International Plant Names Index* (IPNI) (https://www.ipni.org/). *A. rumphianus* was treated as a synonym of *A. calamus*, and all people accepted that it did not represent any independent species (http://www.theplantlist.org/).

*Acorus tatarinowii* has widely been used as herbal medicine in China for 2000 years. Since the first edition, it has been included in the Chinese Pharmacopoeia as the basis plant of traditional Chinese medicine called Shichangpu. It is used for multiple medicinal purposes, particularly for the treatment of central nervous system diseases (Rajput et al., 2014; Sharma et al., 2014). This taxon, however, was treated as a synonym of *A. gramineus* in FOC, or *A. calamus* in IPNI.

*Acorus macrospadiceus*, described as a new species in 1985 by two Chinese botanists, Fa-nan Wei and Yin-kun Li (Wei and Li, 1985), was regarded as a synonym of *A. gramineus* in FOC and IPNI. Interestingly, people in some ethnic groups such as Miao, Dong, She, Shui, Yao, and Tujia in China call it *shan-nai* (in southern China, *shan-nai* refers to *Kaempferia galanga* L., a common spice and medicinal plant in Zingiberaceae) probably because of its smell. The indigenous people believe it represents an independent species different from other *Acorus* plants. It is popular that the local people in ethnic communities use it as a flavoring agent in the preparation of meat and fish. Leaves and rhizomes are both used in cooking (Shu et al., 2018a). However, less study on the taxonomic position of this ethnotaxa had been conducted (Shu et al., 2018b).

In recent years, the medicinal values of *Acorus* were increasingly recognized, and more *Acorus* materials had been collected for medications (Huang et al., 2012; Shu et al., 2018a). Unfortunately, the authorized taxonomic treatment of *Acorus* species confused collectors and consumers with their classification, partly because of their similar morphology, especially when dried. Our recent investigation revealed the volatile oils among different *Acorus* taxa were differed in their oil content and composition based on gas chromatography-mass spectrometry (GC-MS) analysis (Shu et al., 2018b), which supported that *A. macrospadiceus* may be designated as an independent species (Wang et al., 2014). The utilization and medication safety of *Acorus* caused by its unclear taxonomy is increasingly becoming an urgent demand.

Folk taxonomy or parataxonomy, a research field in ethnobiology, is a naming system rooted in traditional culture and can be comparable with scientific taxonomy (Berlin, 1973; Atran, 1998; Wang et al., 2003). Folk taxonomy not only reflects how people describe an organism of a natural ecosystem but also relates it to their local culture (Brown, 1977). Folk taxonomy involves multiple classification mechanisms such as morphology, sensory perception, ecology, and utilitarian characters taxonomy (Ragupathy et al., 2009). After comparison with many examples, anthropologists, linguists, and plant taxonomists believed folk taxonomy should be regarded as a new standard for recording information about organisms, which provides more cultural information about an organism than more scientific systems (Berlin et al., 1966; Breedlove et al., 1968; Raven et al., 1971; Berlin et al., 1973). Yet unlike scientific taxonomy, folk taxonomy is poorly recorded. With the acceleration of urbanization and economic development around the world, fewer dialects are used, leading to a decline in the use of folk taxonomy and its eventual extinction (Galvagne Loss et al., 2014; Poncet et al., 2015; Phaka et al., 2019).

Recently, molecular phylogenetic analyses have widely been used in phylogenetic research, cryptic species identification, traditional medicinal plant authentication, and culture diversity conservation (Hebert et al., 2003; Han et al., 2016; Li et al., 2016; Dong et al., 2017; Dechbumroong et al., 2018; Guo et al., 2018). Researchers have identified some cryptic species and new species by DNA barcoding, and their results were consistent with the folk taxonomic designations of local people (Ragupathy et al., 2009; Newmaster and Ragupathy, 2010; Sui et al., 2011; Gao et al., 2017; Ji et al., 2020).

Chemotaxonomy applies chemical differences to classify plants (Hegnauer, 1986; Li et al., 2017). It is widely used to identify plant species by comparing the secondary metabolites among populations (Guo et al., 2008; Li M. H. et al., 2010). The 13C NMR-based metabolomics was useful for distinguishing the species and origins of green coffee beans from different geographic regions (Wei et al., 2012). The compared genetic and phytochemical taxonomy methods could be used to classify *Rhodiola* species (Liu et al., 2013).

Previous reports showed probably two to four taxa existed in the genus *Acorus* (Li, 1979; Wei and Li, 1985; Li H. et al., 2010; Shu et al., 2018a; Shu et al., 2018b). Thus we proposed a hypothesis that four species occur in *Acorus*, namely *A. calamus* L., *A. gramineus* Aiton, *A. tatarinowii* Schott, and *Acorus macrospadiceus* F. N. Wei et Y. K. Li.

The purpose of this study was to examine the above hypothesis and determine the species number in *Acorus*, and to provide supports for resource management and uses. In this study, we combined multiple methods including phylogenetics and metabolomics, together with ethnobotanical, morphological, and ecological approaches.

## Materials and Methods

### Materials

No matter what opinion adopted by taxonomists and users, all taxa at species level in *Acorus* are distributed in China. Therefore, we collected most samples from China for this study. A total of 82 samples and associated voucher specimens belonging to different *Acorus* taxa were collected from five countries (China, Japan, Portugal, Myanmar, and Bhutan) during field investigations from 2013 to 2016. Samples collected from China covered ten provinces: Chongqing, Guangxi, Guizhou, Heilongjiang, Hubei, Hunan, Jiangxi, Sichuan, Yunnan, and Zhejiang. Samples were gathered from each geographic location. Leaf samples for extracting DNA were collected, silica-dried, and stored at −20°C for laboratory analysis. The information about collection sites, voucher specimen numbers, and GenBank accession numbers were documented (**Supplementary Table S1**). For metabolomic study, 26 samples were collected in four provinces of China, including 20 samples from Guizhou, three samples from Hunan, two samples from Yunnan, and one sample from Beijing (**Supplementary Table S2**). All plant species were identified by Prof. Chunlin Long. The voucher specimens were deposited in the Herbarium of the College of Life and Environmental Sciences, Minzu University of China, in Beijing.

For metabolite isolation, leaves and rhizomes of *Acorus* were naturally dried, powdered with a pulverizer (Sainai, Shanghai, China), and filtered through 40 mesh (screen hole size is 0.425 millimeter) sieve. About 0.2 g of powder samples were transferred into centrifuge tubes, 10 ml of 80% methanol (v/v) was added, blended for 2 min, and extracted using ultrasound for 30 min at room temperature. Samples were allowed to settle for 5 min, centrifuged at 5,000 rpm (Revolutions per minute) for 10 min, and the supernatant was transferred to a new tube. The previous extraction steps were repeated twice by adding 200 µl of 80% methanol each time, and the supernatants were combined. Then the solvent was evaporated under a stream of N^2^ gas at room temperature. The extracts were placed in a refrigerator at −20°C for storage. To validate the analytical method and monitor the stability of the system, a pooled quality control (QC) sample was prepared by mixing the same volume from all samples of *Acorus* in different parts (Gu et al., 2019). Before UPLC-QTOF-MS analysis, the crude extracts were redissolved at a concentration of 1 mg/ml in 80% methanol aqueous solutions, ﬁltered through a 0.45 μm polytetraﬂuoroethylene (PTFE) syringe ﬁlter. The injection volume was 1.0 μl.

### Ethnobotanical Surveys

From 2006 to 2017, the authors visited 15 provinces, regions, or municipalities of China including Anhui, Chongqing, Fujian, Guangxi, Guizhou, Heilongjiang, Hubei, Hunan, Jiangsu, Jiangxi, Sichuan, Taiwan, Tibet, Yunnan, and Zhejiang to gather ethnobotanical information of *Acorus* plants. Informants ranging in age from 14 to 96 years old, including 262 males and 311 females, were interviewed. The 573 informants were from the following linguistic groups: Miao, Yao, Buyi, Shui, Tujia, Dong, She, Maonan, Zhuang, and Yi, as well as the Han Chinese. Ethnobotanical data were collected through semi-structured interviews, key informant interviews, and participatory tools. Informed consent was verbally obtained from all participants prior to the study.

### Morphological and Ecological Investigations

During field surveys, direct and participatory observations were conducted to record the morphological traits and features, habitats, and distribution patterns. Herbarium specimens were collected, and identified tentatively. The important morphological traits such as rhizomes, leaves, inflorescences, and infructescences were measured in the fields. The ecological habits of *Acorus* were investigated through direct observation and semi-structured interviews (if habits changed).

### DNA Isolation, PCR Amplification, Sequencing, and Alignment

Two chloroplast gene fragments (*rbcL*, and *trnL-trnF*) were used to determine genetic relationships of *Acorus*. The CTAB method was applied to extract total DNA of *Acorus* (Murray and Thompson, 1980). The relative purity and concentration of extracted DNA were detected by 1% agarose gel electrophoresis and spectrophotometer. PCR amplification was performed in a 25 µl reaction mixture containing 2.5 µl of 10 × PCR buffer, 2.0 µl of 25 mmol/L MgCL_2_, 2.5 µl of 2.5 mmol/L dNTPs, 0.5 µl of 100 µmol/L each primer, 0.5 unit of Taq DNA polymerase, 1 µl of genomic DNA (~30 ng), and double-distilled water. Amplification and primer of the two molecular genetic markers *rbcL* and *trnL-trnF* was carried out as described in **Supplementary Table S3** (Taberlet et al., 1991; Fay et al., 1997; Kress and Erickson, 2007). The PCR products were detected by 1% agarose gel electrophoresis and observed under the gel imaging system. In order to ensure the accuracy of the data, two-way sequencing of the PCR products that meet the requirements of sequencing were carried out. Purification and sequencing of DNA products were completed by Boshang Biotechnology Co., Ltd. (Beijing). The sequenced peak maps were adjusted manually by Chromos software to remove the low-quality sequences. BLAST search was used to compare with the existing data on NCBI (https://www.ncbi.nlm.nih.gov/) to ensure the accuracy of sequence obtained (Altschul et al., 1997). These sequences were then aligned manually in MEGA X (Kumar et al., 2018), and concatenated in Sequence Martix-1.8. *Kaempferia rotunda* L. (Zingiberaceae) was selected as outgroup of phylogenetic trees. The sequence *Kaempferia rotunda* was downloaded from GenBank (**Supplementary Table S1**).

### Phylogenetic Analyses

Maximum likelihood (ML) and Bayesian inference (BI) methods were used to reconstruct the relationships within *Acorus*. JModelTest v.2.0 (Guindon and Gascuel, 2003; Darriba et al., 2012) was run to select the appropriate model of nucleotide evolution, using the Akaike information criterion (AIC; Akaike, 1974), and the results were the basis of both ML and BI analyses.

The ML analysis was conducted using IQ-TREE version 1.6.2 (Nguyen et al., 2015) with 1,000 rapid bootstrapping replicates and the molecular evolution model (HKY+I) determined by jModelTest. For Bayesian tree inference, two runs each with four chains were done for one million generations using MrBayes v.3.2.6 (Ronquist and Huelsenbeck, 2003). The first 25% of the generations were discarded as burnin to ensure that the likelihood values have reached convergence. The resulting tree was visualized in FigTree 1.4.3 (http://tree.bio.ed.ac.uk/software/figtree/). Bootstrap percentage (BP) values of 70% or higher were considered statistically significant and indicated a well-supported clade and Bayesian posterior probability (BPP) values more than 0.95 were regarded as good support (Hillis and Bull, 1993; Larget and Simon, 1999). In this article, BP ≥ 70 and BPP ≥ 0.90 are shown at branches.

### Metabolite Analysis by UPLC-QTOF-MS

UPLC analysis was operated by ACQUITY UPLC systems (Waters, Milford, MA, USA) coupled with QTOF-MS (Xevo 2 QTOF, Waters MS Technologies, Manchester, UK), controlled by Masslynx 4.1 (version 4.1, Waters, Milford, MA, USA).

MS full scan mode and MS^E^ model was adopted. The mass detection range of the full-scan analysis mode was 100–1,000 Da for m/z, 3 kV for normal capillary, 2.5 kV for negative capillary, 1 s for scanning times, 800 and 50 L/h for desolvation and cone gas for N_2_, respectively. The ion source temperature and desolvation temperatures were 110°C and 400°C for MS^E^ analysis. The quality detection range, N_2_ flow rate, source temperature, and desolvation temperature of the model are identical to those of the MS mode. Normal analysis was in the Centroid mode, the scanning time was 1 s, the low energy voltage was 6 V, and the high energy voltage was 20–60 V. The A solution of Leucine Enkephalin (Sigma Chemical, L9133) with a flow rate 20 ugL/min was used in the Lock-Spray to obtain accurate mass measurements. The Lock mass of the positive and negative state analysis was m/z 556.2771 and m/z 554.2615, respectively.

Chromatographic separation was performed with an inner diameter, UPLC BEH C18 reversed-phase column (2.1 × 50 mm, i.d. 1.7 μm Waters Corp., Milford, MA, USA) maintained at 40°C. The mobile phase consisted of 0.1% aqueous formic acid (A) and 0.1% formic acid in acetonitrile (B). The linear gradient elution was performed as follows: 0–4.5 min, 0–3% B; 4.5–6 min, 3–95% B; 6–6.5 min, 95-% B; 6.5–8 min, 3-3% B. The ﬂow rate was 0.5 ml/min and the injection volume 1.0 μl.

### Multivariate Analysis

The chromatographic-mass raw data in the positive ion mode were preprocessed by MarkerLynx XS including peak detection, peak alignment, peak integration, and the retention time correction. In order to understand similarities and differences of metabolic components among species, principal component analysis (PCA) was based on mass spectrometry data obtained from UPLC-QTOF-MS full scan mode in the MarkerLynx XS v4.1 software. Heat-map analysis and hierarchical clustering analysis (HCA) were performed with Heml software to identify homogeneous clusters based on the peak area of characteristic metabolic components and illustrate content changes of metabolites in different groups.

## Results

### Parataxonomy and Ethnobotany of *Acorus*

The ethnobotanical survey documented that 29 ethnic groups (including the Han Chinese) use *Acorus* plants for culture, edible, medicinal, and ornamental use in China. People of different linguistic groups in southwestern and central China have a lot of traditional knowledge to classify and use different taxa of *Acorus*. In Qiandongnan Prefecture of Guizhou Province, local people such as Miao (including Gejia and Chuanqing branches), Dong, Buyi, She, Yao, and Han can recognize four different taxa in the genus *Acorus*. They adopted growth status, morphology, habit, and smell in their folk taxonomy (**Table 1**). In particular, they identified *A. macrospadiceus* easily based on these features, although it is very similar to *A. tatarinowii* morphologically. Different taxa were used for different purposes by local people in Qiandongnan (**Table 1**).

**Table 1:** Morphological, ecological, and ethnobotanical information of four *Acorus* species.

Species	Local name*	Chinese name	Morphology	Habitat	Smell	Traditional use*
*A. calamus*	Jiabao-weng, Bota	Changpu	Rhizome white, 5–25 × 1–4 cm. Leaves with a distinct midrib; ensiform, upward, 90–150 × 1–3 cm	Growing in mud of swamps, pond sides, standing water; alt. < 2,800 m	Rhizome: aromatic	Rhizome for medicine; Whole plant for cultural purpose
*A. tatarinowii*	Jiabu-wu, Lubu-wei	Shichangpu	Rhizome green, 5–15 × 0.5–1 cm. Leaves without midrib; drooping, 20–50 × 0.7–1.3cm	Epiphyte on rocks or rocky banks of brooks with fast-flowing water; alt. < 2,600 m	Rhizome: aromatic	Rhizome for medicine
*A. gramineus*	Wendeng-bo	Jinqianpu	Rhizome dark green, 3–8 × 0.4–0.6 cm. Leaves without midrib; linear, drooping, 20–30 × < 0.7 cm	Growing in forests or on stream banks; < 1,800 m	Rhizome: aromatic	Rhizome for medicine; Whole plant as ornamental
*A. macrospadiceus*	Shan-nai, Gourou-xiang, Xiang nai	Huixiang Changpu	Rhizome dark green, 8–20 × 0.7–1.2 cm. Leaves without midrib; drooping, 30–85 × 0.7–1.5 cm	Growing on mountain slopes with wet soil or brook banks; < 1,500 m	Whole plant: strong aromatic	Whole plant as spice for cooking

*Taking Qiandongnan Prefecture of Guizhou Province as an example.

### Morphological and Ecological Characteristics of Different *Acorus* Species

The differences of rhizome size and leaf length among various taxa were recorded in **Table 1**. *Acorus calamus* and *A. gramineus* can be easily separated from each other for their morphological differences. But it is difficult to differentiate *A. tatarinowii* from *A. macrospadiceus* for their morphological similarities.

The ecological habits varied in different species. *A. calamus* and *A. tatarinowii* are aquatic plants, while *A. gramineus* and *A. macrospadiceus* are terrestrial taxa. *A. calamus* is an emergent plant and grows in calm water, slowly flowing water, or aquatic environment. *A. tatarinowii* is an epiphytic plant on rocks in fast-flowing brooks. *A. gramineus* grows in drier soil with lower humidity, and *A. macrospadiceus* grows in wet soil with higher humidity (**Table 1**).

### Molecular Phylogeny

**Figure 1:**
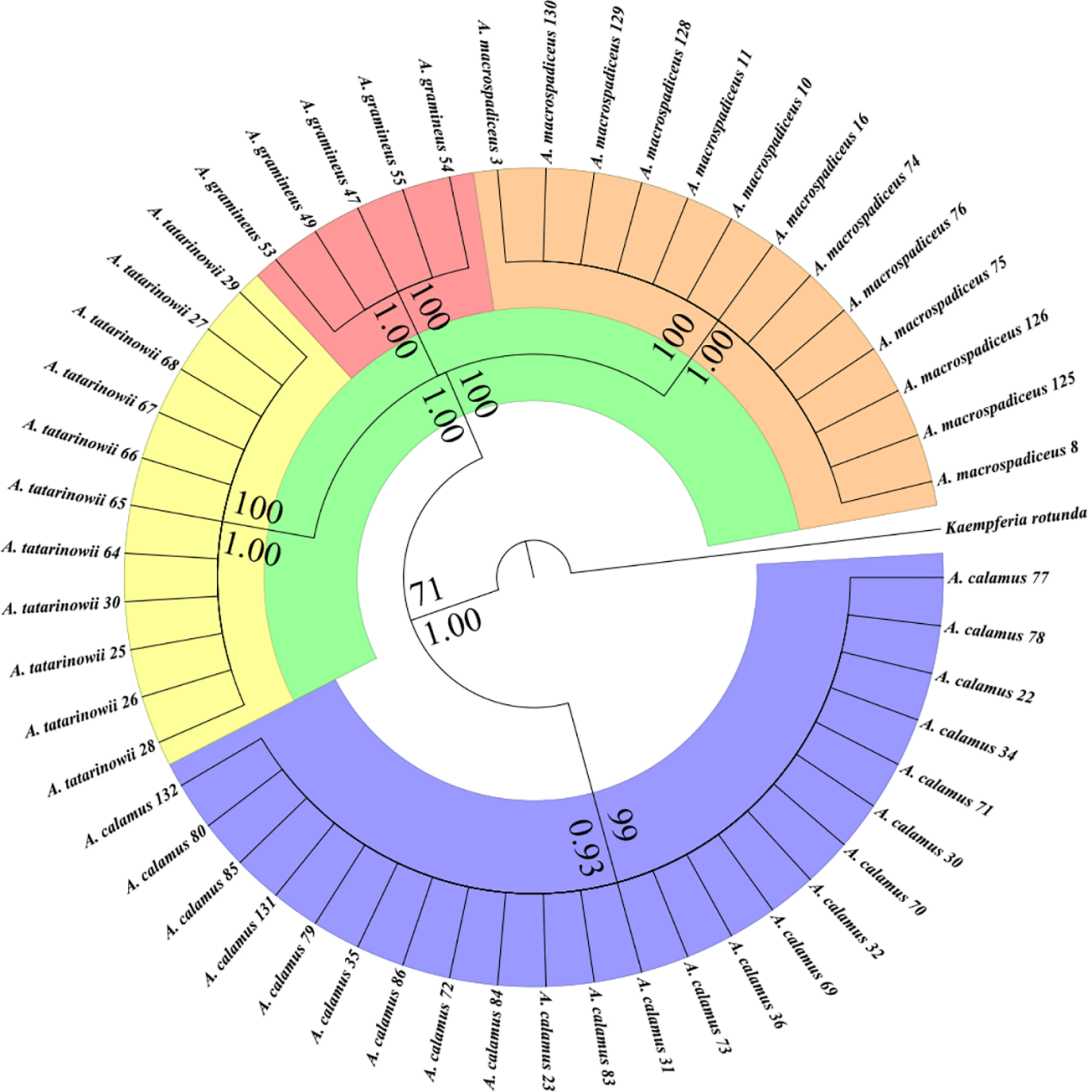
Phylogeny of *Acorus* inferred with the maximum likelihood and Bayesian analyses based on two markers from chloroplast DNA. Bootstrap percentage (BP) ≥70 and Bayesian Posterior Probability (BPP) ≥0.90 are shown at branches. Four species gathered to different clusters and occupied independent branches. Blue, *A. calamus*. Red, *A. gramineus*. Orange, *A. macrospadiceus*. Yellow, *A. tatarinowii*.

The Bayesian and Maximum Likelihood tree topologies of *Acorus* are identical (**Figure 1**). *Acorus* splits into two well supported main clades, one clade is *A. calamus* (BP 99, BPP 0.93) covered with blue and the other clade conclude three species (BP 71, BPP 1.00) cover with green. In this three species clade, *A. gramineus* (BP 100, BPP 1.00) cover with red, *A. tatarinowii* (BP 100, BPP 1.00) cover with yellow and *A. macrospadiceus* (BP 100, BPP 1.00) cover with orange. Four species gathered to different clusters and occupied independent branches in phylogenetic trees. Based on our molecular phylogeny investigation, we think *A. tatarinowii* and *A. macrospadiceus* should be treated as an independent species of *Acorus* (**Supplementary Figures S2** and **S3**).

### Multivariate PCA of Different Parts of *Acorus*

**Figure 2:**
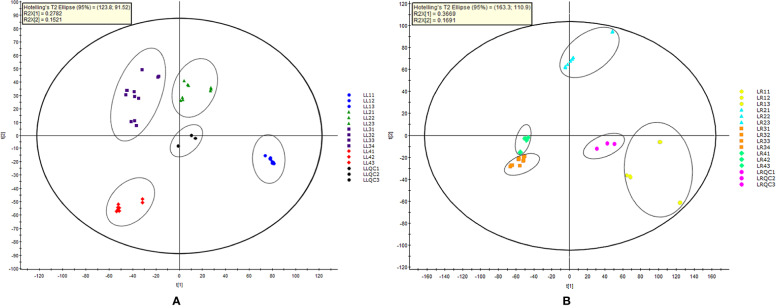
Principal component analysis score plot of different parts (**A**, leaf samples; and **B**, rhizome samples) of *Acorus*. **LL**, leaf simple; **LR**, rhizome simple; LL11–LL13 and LR11–LR13, *A. calamus*; LL21–LL23 and LR21–LR23, *A. tatarinowii*; LL31–LL34 and LR31–LR34, *A. macrospadiceus*; LL41–LL43 and LR41–LR43, *A. gramineus*; LLQC1–LLQC3, leaf sample quality control; LRQC1–LRQC3, rhizome sample quality control.

UPLC-QTOF-MS was performed on extracts from four *Acorus* taxa, and each sample was injected three times. The retention time (RT) of the main compounds, the ion peak, and main fragment peaks of the mass spectrum were recorded. In order to understand the similarities and differences of metabolic components among *Acorus* species, PCA was conducted on mass spectrometry data obtained under UPLC-QTOF-MS in the full scan mode (Wu et al., 2013). MarkerLynx XS v4.1 software generated score plots respectively for PCA of leaf and rhizome extracts from different *Acorus* taxa (**Figures 2A, B**).

As shown in **Figure 2A**, the first and second components explained 27.8 and 15.2% of the total variance, respectively. Thirteen samples were divided into four clusters, and the same species from different populations clustered together. In **Figure 2B**, the first and second principal components explained 36.7 and 16.9% of the total variance, respectively. Similarly, the results confirmed that *Acorus* taxa were divided into four clusters, indicating significant differences in secondary metabolites of leaves and rhizomes for each taxon.

### Analysis of Main Metabolic Components in Different Parts of *Acorus*

Characteristic compounds of *Acorus* species were selected through PCA. These characteristic compounds have important taxonomic and phylogenic significance in phytochemical taxonomy. In the load matrix of PCA, we found the contribution of variables to the classification of different groups. The greater the contribution coefficient value is, the bigger the effect of that individual variables (i.e. metabolites) on classification will be.

Generally, if the retention time-mass (RT-MS) is farther away from the center, the contribution of the RT-EM will be bigger. These greater contribution RT-EMs were the main metabolites characterized. Therefore, according to the load matrix, metabolites were characterized for different *Acorus* species.

Variable importance in the project (VIP) value reflects the influence of every metabolite ion on classification, variables with a VIP >1 indicated a significant contribution to the separation of a sample group (Chen et al., 2009). Therefore, metabolites with a VIP value >4.897 were selected for further study, and 39 RT-MS compounds were selected as potential markers to distinguish species.

All UPLC-QTOF-MS data (normal) were included in the relative content analysis of metabolites. The peak areas of these 39 potential marker metabolites were analyzed by Quanlynx software for heat-map analysis (**Supplementary Figure S1** and **Supplementary Table 4**).

### Heat Map and HCA of Different Parts of *Acorus*

**Figure 3:**
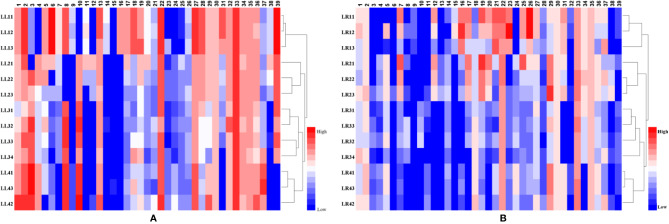
The heat-map and HCA of differential metabolites from different parts (**A**, leaf samples; and **B**, rhizome samples) of *Acorus*. LL, leaf sample; LR, rhizome sample; LL11–LL13 and LR11–LR13, *A. calamus*; LL21–LL23 and LR21–LR23, *A. tatarinowii*; LL31–LL34 and LR31–LR34, *A. macrospadiceus*; LL41–LL43 and LR41–LR43, *A. gramineus*.

To visualize chemical differences among four species of *Acorus*, a heat map of 13 samples for 39 metabolites in the negative ion mode was plotted (**Figure 3**). The red and blue colors in the plot represent higher and lower metabolite contents compared with mean values, respectively.

HCA is an exploratory analysis that classifies variables by clustering samples with similar features, and separating samples with different features (Kress et al., 2005). To observe metabolite cluster patterns of *Acorus* samples, HCA of leaf and rhizome tissues were performed. All samples were clustered into four categories (**Figures 3A, B**). In the HCA of leaf samples, *A. macrospadiceus*, *A. tatarinowii*, and *A. gramineus* clustered with *A. calamus*. It is obvious that *A. macrospadiceus* is different from the other three species, but has the highest similarity and the closest genetic relationship with *A. gramineus* (**Figure 3A**). Similar HCA results were obtained for metabolic profiling of rhizome extracts (**Figure 3B**).

## Discussions

### Traditional Uses of *Acorus* in China

*Acorus* plants are traditionally used for cultural, edible, medicinal, and ornamental purposes. The meaning of “changpu” is a general name for *Acorus* in Chinese. In some linguistic groups, they are also used to clean up water, and repel mosquitoes (Shu et al., 2018a). All *Acorus* species have been used as medicinal materials. Both *A. tatarinowii* (Shichangpu) and *A. calamus* (Changpu) are common herbal medicinal plants in different cultures. Their most commonly used parts are rhizomes which are widely utilized to treat various ailments. Their whole plants, leaves, and inflorescences are also used as medicines. We found, for example, that the Mulao (Mulam) people collected their inflorescences to treat wounds. *A. gramineus* (Jinqianpu) and *A. macrospadiceus* (Shannai) were used as a flavoring agent in the preparation of meat and fish in Guizhou, Chongqing, Guangxi, and surrounding areas. Shannai is more popular in local diets. The local people regarded *Acorus* plants as a sacred and symbolic plant, because the leaves of the *A. calamus* look like swords and they have super natural power to exorcise evil spirits. Culturally *A. calamus* (Changpu) is the most frequently used species on the Dragon Boat Festival. People hang the whole plant on the top of their front doors or main gates together with *ai* (*Artemisia argyi*) to keep evil away from the house and family, and they pray for safety for anyone taking trips outside the house. *A. gramineus* was often used for indoor bonsai. In some areas we studied, the local people cultivated *A. calamus* or *A. tatarinowii* to clean up water. Yao and Miao used *A. calamus* as an insect repellent. The dry leaves of *A. calamus* had been used for tying *zongzi* (a traditional food made from sticky rice and other foodstuff packed by bamboo leaves) (Shu et al., 2018a).

### Acorus macrospadiceus and A. tatarinowii, Two Independent Species

In this study, multiple evidences including those from morphology, ecology, folk classification, phylogenetic, and chemical taxonomy were obtained to examine relationships among *Acorus* species. In many ethnic communities of southwest China, people considered *A. macrospadiceus* as an independent species differing from others (*A. calamus*, *A. gramineus*, and *A. tatarinowii*). It is different from the taxonomic treatment in FOC, IPNI, or the *Plant List* in which *A. macrospadiceus* was incorporated into *A. gramineus*. The results of phylogenetics and chemotaxonomy showed an obvious distinction between *A. macrospadiceus* and other *Acorus* species (**Figures 1**–**3**), which is consistent with the view of folk taxonomy. Our results oppose regarding *A. macrospadiceus* as a synonym for *A. gramineus*. We confirmed the species of *Acorus macrospadiceus* (Yamamoto) F. N. Wei et Y. K. Li.

Our research result also opposed that *Acorus tatarinowii* was regarded as a synonym of *A. gramineus* in FOC or *A. calamus* in IPNI. We support the confirmation of *Acorus tatarinowii* Schott. As one of the oldest Chinese *materia medica* recorded in ancient literatures and accepted by all editions of *Chinese Pharmacopeia*, *A. tatarinowii* has been in a very important position in traditional Chinese medicine and ethnomedicine for decades. Our confirmation of this species will strongly support the traditional medicinal uses and its pharmacy industry development of *A. tatarinowii*.

### Authentication of *Acorus* Species for Different Medicinal Uses

During our field investigations, we found that herbal medicinal merchants often purchased *Acorus* plants from local people. Some vendors confused *A. macrospadiceus* with *A. tatarinowii*. They collected *A. macrospadiceus* massively to sell as a medicinal plant but not as a spice, which may result in over-harvest.

More significantly, the misuse or abuse of wrong *Acorus* species may threaten the safety in clinics because chemical constituents of different taxa vary species by species. Our previous research illustrated that the volatile oil from both rhizomes and leaves of *A. macrospadiceus* primarily contains estragole, β-caryophyllene, trans-anethole, β-elemene, endo-borneol, and other volatile ingredients. Estragole is the main component (Shu et al., 2018b). Differently, it was reported that the main volatile components of *A. tatarinowii* were α-asarone, β-asarone, and γ-asarone through GC-MS analysis, with β-asarone being presented in the highest quantities (Jaiswal et al., 2015). This illustrates that attention should be paid to differentiate *A. tatarinowii* and *A. macrospadiceus* for medicinal uses. To identify *Acorus* species correctly and precisely will assist the drug administration to issue policies and regulations for quality control. The authentication of *Acorus* species becomes great significance for medicinal uses of the genus.

### Conservation of Folk Taxonomy

Various studies based on multiple ethno-disciplines (folk avian taxonomy, folk insect taxonomy, folk plant taxonomy) have proven that folk taxonomy is consistent with scientific taxonomy (Raven et al., 1971; Mourão et al., 2006; Ramires et al., 2012). In modern plant taxonomy, most of the folk taxonomies had been ignored. The acceleration of urbanization and impacts from mainstream culture have led to the reduction and even extinction of parataxonomy. The case of *Acorus* showed traditional botanical knowledge is decreasing, especially in areas with rapid economic development. For example, less and less people collected *Acorus* species to treat various diseases, to compare with three decades ago. The young people prefer to stay in the urban areas, and are not familiar with traditional knowledge of *Acorus*. Few people less than 20 years old could recognize different *Acorus* species, according to our ethnobotanical investigations. In recent years, there are many studies on the chemical components and pharmacodynamics of *Acorus*, which prove the rationality of their traditional uses. Unfortunately, indigenous people are rapidly losing their traditional botanical knowledge including folk taxonomy. This study illustrates the validity of parataxonomy for identifying plant resources used among traditional communities.

## Conclusion

We verified our hypothesis that four species occurred in the genus *Acorus* (Acoraceae) based on multiple approaches including phylogenetics, metabolomics, morphology, ecology, and ethnobotany as well. They are *Acorus calamus* L., *Acorus gramineus* Aiton, *Acorus macrospadiceus* (Yamam.) F. N. Wei & Y. K. Li, and *Acorus tatarinowii* Schott. Our research results supported the existence of an ethnotaxa, *A. macrospadiceus*, and elucidated the significance of folk taxonomy. The confirmation of the most important medicinal species in the genus, *A. tatarinowii* or Shichangpu in Chinese, provided essential basis for quality control and pharmacy development of *Acorus*. The conservation of *Acorus* resources and associated traditional knowledge was also suggested in the present paper.

## Data Availability Statement

The original contributions presented in the study are included in the article/**Supplementary Material**. Further inquiries can be directed to the corresponding author.

## Author Contributions

CL conceived and designed the experiments. ZC, HS, SZ, BL, RG, RZ, YJ, and FL performed the experiments and collected the data. ZC, HS, and SZ analyzed and uploaded the data. ZC wrote the manuscript. BL, RG, RZ, and CL revised the manuscript and edited the language. All authors contributed to the article and approved the submitted version.

## Funding

This work was supported by grants from the National Natural Science Foundation of China (No. 31761143001, 31870316), Biodiversity Survey and Assessment Project of the Ministry of Ecology and Environment of China (2019HJ2096001006), Key Laboratory of Ethnomedicine (Minzu University of China) of Ministry of Education of China (KLEM-ZZ201904 & KLEM-ZZ201906), Jiansheng Fresh Herb Medicine R & D Foundation (JSYY-20190101-043), Minzu University of China (Collaborative Innovation Center for Ethnic Minority Development and YLDXXK201819), and Ministry of Education of China and State Administration of Foreign Experts Affairs of China (B08044).

## Conflict of Interest

The authors declare that the research was conducted in the absence of any commercial or financial relationships that could be construed as a potential conflict of interest.
