# Supplementary figures and images for: From Folk Taxonomy to Species Confirmation of Acorus (Acoraceae): Evidences Based on Phylogenetic and Metabolomic Analyses
Source: Front Plant Sci. 2020 Jun 24;11:965. doi: 10.3389/fpls.2020.00965 (PMC7327505; doi:10.3389/fpls.2020.00965)

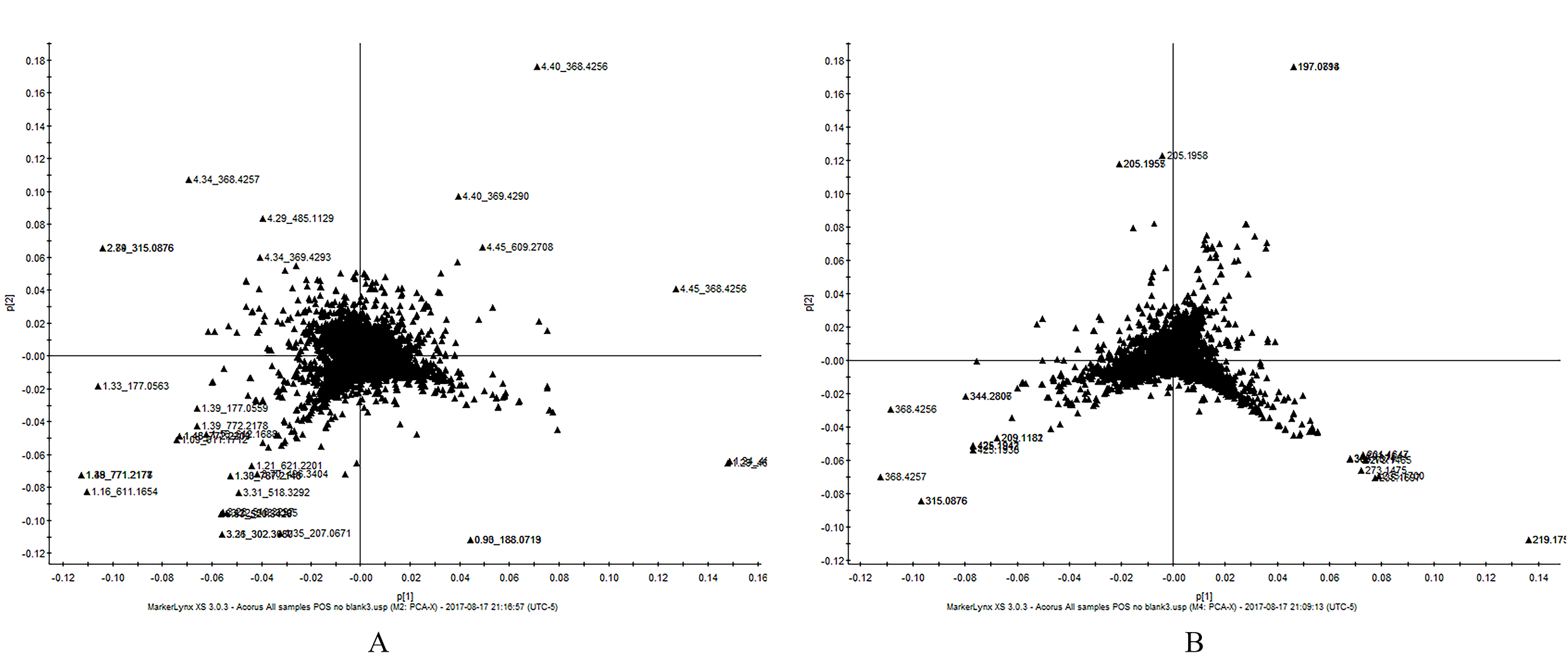

Supplement: Supplementary file 1 [file Image_1.tif]

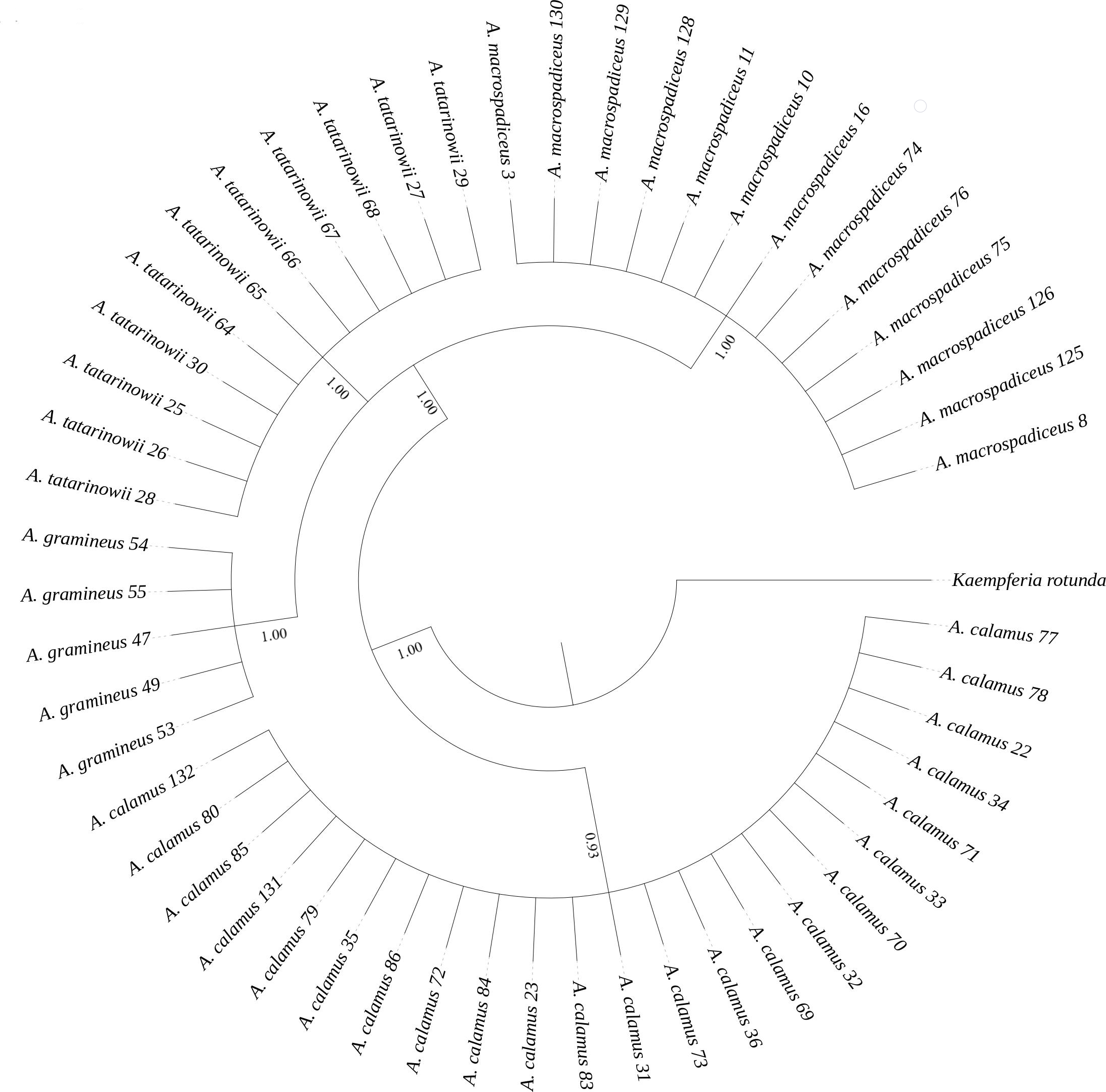

Supplement: Supplementary file 2 [file Image_2.tif]

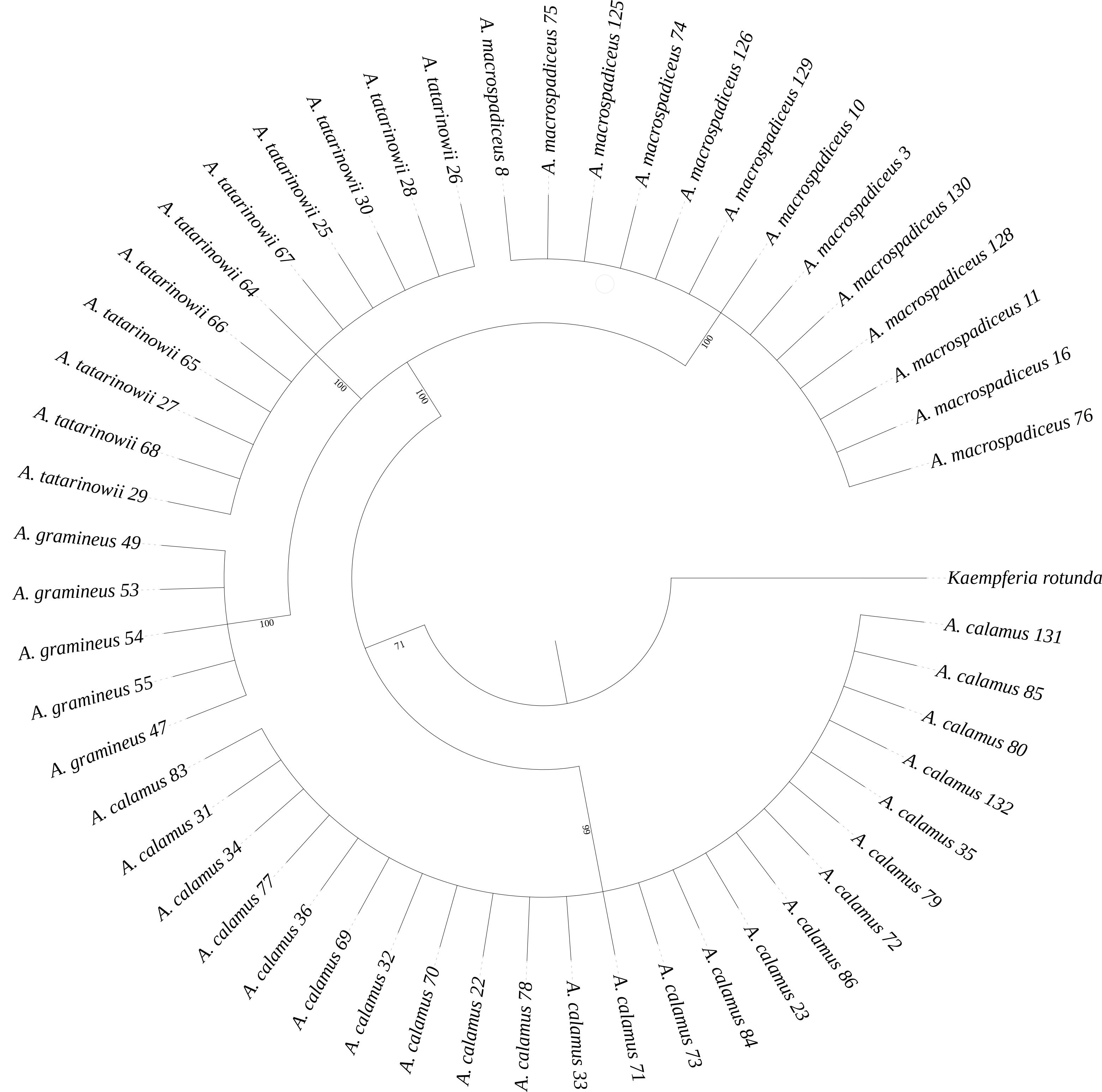

Supplement: Supplementary file 3 [file Image_3.tif]
