# Supplementary material for: From Folk Taxonomy to Species Confirmation of Acorus (Acoraceae): Evidences Based on Phylogenetic and Metabolomic Analyses
Source: Front Plant Sci. 2020 Jun 24;11:965. doi: 10.3389/fpls.2020.00965 (PMC7327505; doi:10.3389/fpls.2020.00965)
Supplement: Supplementary file 4 [file Table_1.docx]

**Table S1 |** Voucher information, sample origin, and GenBank accession numbers for *Acorus* plant samples in this study.

| **Sample** | **Sample origin** | **Voucher Number** | **GenBank accession number** | |
| --- | --- | --- | --- | --- |
|  |  |  | ***rbcL*** | ***trnL-trnF*** |
| *Acorus. macrospadiceus* | Qiandongnan Guizhou China | SN01 | —— | MN489326 |
| *Acorus. macrospadiceus* | Qiandongnan Guizhou China | SN02 | —— | MN489327 |
| *Acorus. macrospadiceus* | Qiandongnan Guizhou China | SN03 | MN489270 | MN489328 |
| *Acorus. macrospadiceus* | Qiandongnan Guizhou China | SN05 | —— | MN489329 |
| *Acorus. macrospadiceus* | Qiandongnan Guizhou China | SN06 | —— | MN489330 |
| *Acorus. macrospadiceus* | Qiandongnan Guizhou China | SN07 | —— | MN489334 |
| *Acorus. macrospadiceus* | Kaili Guizhou China | SN08 | MN489271 | MN489335 |
| *Acorus. macrospadiceus* | Hechi Guangxi China | SN09 | —— | MN489336 |
| *Acorus. macrospadiceus* | Qiannan Guizhou China | SN10 | MN489278 | MN489306 |
| *Acorus. macrospadiceus* | Qiannan Guizhou China | SN11 | MN489279 | MN489314 |
| *Acorus. macrospadiceus* | Qiandongnan Guizhou China | SN16 | MN489280 | MN489325 |
| *Acorus. macrospadiceus* | Jiujiang Jiangxi China | SN74 | MN489281 | MN489331 |
| *Acorus. macrospadiceus* | Jiujiang Jiangxi China | SN75 | MN489282 | MN489332 |
| *Acorus. macrospadiceus* | Jiujiang Jiangxi China | SN76 | MN489283 | MN489333 |
| *Acorus. macrospadiceus* | Shizhu Chongqing China | SN101 | —— | MN489301 |
| *Acorus. macrospadiceus* | Shizhu Chongqing China | SN102 | —— | MN489302 |
| *Acorus. macrospadiceus* | Shizhu Chongqing China | SN103 | —— | MN489303 |
| *Acorus. macrospadiceus* | Shizhu Chongqing China | SN108 | —— | MN489304 |
| *Acorus. macrospadiceus* | Shizhu Chongqing China | SN109 | —— | MN489305 |
| *Acorus. macrospadiceus* | Shizhu Chongqing China | SN110 | —— | MN489307 |
| *Acorus. macrospadiceus* | Shizhu Chongqing China | SN111 | —— | MN489308 |
| *Acorus. macrospadiceus* | Shizhu Chongqing China | SN112 | —— | MN489309 |
| *Acorus. macrospadiceus* | Xiushan Chongqing China | SN113 | —— | MN489310 |
| *Acorus. macrospadiceus* | Xiushan Chongqing China | SN114 | —— | MN489311 |
| *Acorus. macrospadiceus* | Xiushan Chongqing China | SN115 | —— | MN489312 |
| *Acorus. macrospadiceus* | Xiushan Chongqing China | SN119 | —— | MN489313 |
| *Acorus. macrospadiceus* | Xiushan Chongqing China | SN120 | —— | MN489315 |
| *Acorus. macrospadiceus* | Xiushan Chongqing China | SN121 | —— | MN489316 |
| *Acorus. macrospadiceus* | Xiushan Chongqing China | SN122 | MN489272 | —— |
| *Acorus. macrospadiceus* | Xiushan Chongqing China | SN123 | —— | MN489317 |
| *Acorus. macrospadiceus* | Xiushan Chongqing China | SN124 | —— | MN489318 |
| *Acorus. macrospadiceus* | Hechi Guangxi China | SN125 | MN489273 | MN489319 |
| *Acorus. macrospadiceus* | Qiannan Guizhou China | SN126 | MN489274 | MN489320 |
| *Acorus. macrospadiceus* | Qiannan Guizhou China | SN127 | —— | MN489321 |
| *Acorus. macrospadiceus* | Qiannan Guizhou China | SN128 | MN489275 | MN489322 |
| *Acorus. macrospadiceus* | Qiannan Guizhou China | SN129 | MN489276 | MN489323 |
| *Acorus. macrospadiceus* | Kaili Guizhou China | SN130 | MN489277 | MN489324 |
| *Acorus. calamus* | Qiandongnan Guizhou China | CP22 | MN481070 | MN481085 |
| *Acorus. calamus* | Changsha Hunan China | CP23 | MN481071 | MN481086 |
| *Acorus. calamus* | Kengtung Myanmar | CP31 | MN481072 | MN481087 |
| *Acorus. calamus* | Kengtung Myanmar | CP32 | MN481073 | MN481088 |
| *Acorus. calamus* | Kengtung Myanmar | CP33 | MN481074 | MN481089 |
| *Acorus. calamus* | Chongqing China | CP34 | MN481075 | MN481090 |
| *Acorus. calamus* | Chongqing China | CP35 | MN481076 | MN481091 |
| *Acorus. calamus* | Chongqing China | CP36 | MN481077 | MN481092 |
| *Acorus. calamus* | Jiujiang Jiangxi China | CP69 | MN481078 | MN481093 |
| *Acorus. calamus* | Jiujiang Jiangxi China | CP70 | MN481079 | MN481094 |
| *Acorus. calamus* | Jiujiang Jiangxi China | CP71 | MN481080 | MN481095 |
| *Acorus. calamus* | Jiujiang Jiangxi China | CP72 | MN481081 | MN481096 |
| *Acorus. calamus* | Jiujiang Jiangxi China | CP73 | MN481082 | MN481097 |
| *Acorus. calamus* | Paro Bhutan | CP77 | MN481058 | MN481098 |
| *Acorus. calamus* | Paro Bhutan | CP78 | MN481059 | MN481099 |
| *Acorus. calamus* | Paro Bhutan | CP79 | MN481060 | MN481100 |
| *Acorus. calamus* | Chengdu Sichuan China | CP80 | MN481061 | MN481101 |
| *Acorus. calamus* | Chengdu Sichuan China | CP81 | MN481062 | —— |
| *Acorus. calamus* | Wudalianchi Heilongjiang China | CP82 | MN481063 | —— |
| *Acorus. calamus* | Beian Heilongjiang China | CP83 | MN481064 | MN481102 |
| *Acorus. calamus* | Xishuangbanna Yunnan China | CP84 | MN481065 | MN481103 |
| *Acorus. calamus* | Xishuangbanna Yunnan China | CP85 | MN481066 | MN481104 |
| *Acorus. calamus* | Xishuangbanna Yunnan China | CP86 | MN481067 | MN481105 |
| *Acorus. calamus* | Zhuji Zhejiang China | CP88 | —— | MN481106 |
| *Acorus. calamus* | Toyama-ken Japan | CP131 | MN481068 | MN481083 |
| *Acorus. calamus* | Okayama-ken Japan | CP132 | MN481069 | MN481084 |
| *Acorus. gramineus* | Kunming Yunnan China | JQP47 | MN489252 | MN489284 |
| *Acorus. gramineus* | Kunming Yunnan China | JQP48 | —— | MN489285 |
| *Acorus. gramineus* | Kunming Yunnan China | JQP49 | MN489253 | MN489286 |
| *Acorus. gramineus* | Porto Portugal | JQP53 | MN489254 | MN489287 |
| *Acorus. gramineus* | Porto Portugal | JQP54 | MN489255 | MN489288 |
| *Acorus. gramineus* | Porto Portugal | JQP55 | MN489256 | MN489289 |
| *Acorus. tatarinowii* | Chenzhou China Hunan | SCP25 | MN489257 | MN489290 |
| *Acorus. tatarinowii* | Chenzhou China Hunan | SCP26 | MN489258 | MN489291 |
| *Acorus. tatarinowii* | Chenzhou China Hunan | SCP27 | MN489259 | MN489292 |
| *Acorus. tatarinowii* | Chenzhou Hunan China | SCP28 | MN489260 | MN489293 |
| *Acorus. tatarinowii* | Chenzhou Hunan China | SCP29 | MN489261 | MN489294 |
| *Acorus. tatarinowii* | Chenzhou Hunan China | SCP30 | MN489262 | MN489295 |
| *Acorus. tatarinowii* | Fangchenggang Guangxi China | SCP44 | MN489263 | —— |
| *Acorus. tatarinowii* | Fangchenggang Guangxi China | SCP45 | MN489264 | —— |
| *Acorus. tatarinowii* | Ganzhou Jiangxi China | SCP64 | MN489265 | MN489296 |
| *Acorus. tatarinowii* | Ganzhou Jiangxi China | SCP65 | MN489266 | MN489297 |
| *Acorus. tatarinowii* | Ganzhou Jiangxi China | SCP66 | MN489267 | MN489298 |
| *Acorus. tatarinowii* | Ganzhou Jiangxi China | SCP67 | MN489268 | MN489299 |
| *Acorus. tatarinowii* | Ganzhou Jiangxi China | SCP68 | MN489269 | MN489300 |
| *Kaempferia rotunda L.* |  |  | GU180520 | GU180520 |
