# Supplementary material for: From Folk Taxonomy to Species Confirmation of Acorus (Acoraceae): Evidences Based on Phylogenetic and Metabolomic Analyses
Source: Front Plant Sci. 2020 Jun 24;11:965. doi: 10.3389/fpls.2020.00965 (PMC7327505; doi:10.3389/fpls.2020.00965)
Supplement: Supplementary file 5 [file Table_2.docx]

**Table S****2 |** Sample part, place of collection, voucher number for *Acorus* plant sample in metabonomic

| **Sample** | **Sample part** | **Place of collection** | **Voucher Number** |
| --- | --- | --- | --- |
| *Acorus. calamus* | leaf | Beijing China | LL11 |
| *Acorus. calamus* | leaf | Hengyang Hunan China | LL12 |
| *Acorus. calamus* | leaf | Yuxi Yunnan China | LL13 |
| *Acorus. tatarinowii* | leaf | Qiandongnan Guizhou China | LL21 |
| *Acorus. tatarinowii* | leaf | Tongren Guizhou China | LL22 |
| *Acorus. tatarinowii* | leaf | Shaoyang Hunan China | LL23 |
| *Acorus. macrospadiceus* | leaf | Qiandongnan Guizhou China | LL31 |
| *Acorus. macrospadiceus* | leaf | Qiandongnan Guizhou China | LL32 |
| *Acorus. macrospadiceus* | leaf | Qiandongnan Guizhou China | LL33 |
| *Acorus. macrospadiceus* | leaf | Kaili Guizhou China | LL34 |
| *Acorus. gramineus* | leaf | Qiandongnan Guizhou China | LL41 |
| *Acorus. gramineus* | leaf | Kaili Guizhou China | LL42 |
| *Acorus. gramineus* | leaf | Qiandongnan Guizhou China | LL43 |
| *Acorus. calamus* | rhizome | Hengyang Hunan China | LR11 |
| *Acorus. calamus* | rhizome | Yuxi Yunnan China | LR12 |
| *Acorus. calamus* | rhizome | Kaili Guizhou China | LR13 |
| *Acorus. tatarinowii* | rhizome | Qiandongnan Guizhou China | LR21 |
| *Acorus. tatarinowii* | rhizome | Qiandongnan Guizhou China | LR22 |
| *Acorus. tatarinowii* | rhizome | Tongren Guizhou China | LR23 |
| *Acorus. macrospadiceus* | rhizome | Qiangdongnan Guizhou China | LR31 |
| *Acorus. macrospadiceus* | rhizome | Qiangdongnan Guizhou China | LR32 |
| *Acorus. macrospadiceus* | rhizome | Qiangdongnan Guizhou China | LR33 |
| *Acorus. macrospadiceus* | rhizome | Kaili Guizhou China | LR34 |
| *Acorus. gramineus* | rhizome | Kaili Guizhou China | LR41 |
| *Acorus. gramineus* | rhizome | Qiandongnan Guizhou China | LR42 |
| *Acorus. gramineus* | rhizome | Qiandongnan Guizhou China | LR43 |
