# Supplementary material for: From Folk Taxonomy to Species Confirmation of Acorus (Acoraceae): Evidences Based on Phylogenetic and Metabolomic Analyses
Source: Front Plant Sci. 2020 Jun 24;11:965. doi: 10.3389/fpls.2020.00965 (PMC7327505; doi:10.3389/fpls.2020.00965)
Supplement: Supplementary file 6 [file Table_3.docx]

**Table S3.** Name of primer and primer sequence used in the study

| *Locus | Primer | Primer sequence (5′–3′) | Reference |
| --- | --- | --- | --- |
| *rbcL* | 1F  724R | ATGTCACCACAAACAGAAACT  TCGCATGTACCTGCAGTAGC | Kress and Erickson, 2007  Fay et al. 1997 |
| *trnL-trnF* | TrnL-F  TrnF-R | CGA AAT CGG TAG ACG CTA CG  GGG GAT AGA GGG ACT TGA AC | Taberlet et al. 1991  Taberlet et al. 1991 |

*rbcL: ribulose-1,4-bisphosphate carboxylase/oxygenase large subunit; trnL-F: trnL gene and trnL-F intergenic spacer.
